# General-purpose AI as a source of mental health support amongst 24,000 adolescents in England

**DOI:** 10.1038/s44184-026-00254-x

**Published:** 2026-10-27

**Authors:** Hannah Okechukwu, Holly Bear, Mina Fazel, Emma Soneson

**Affiliations:** Department of Psychiatry, University of Oxford, Oxford, UK

**Keywords:** Health care, Medical research, Psychology, Psychology

## Abstract

We explored adolescents’ use and experiences of general-purpose AI/LLM platforms for mental health support in a community sample of 24,132 students in England. Overall, 2.7% (*n* = 662) reported past-year use, with 55.9% (324/580) rating AI/LLM support as ‘helpful’. Most (92.1%; 610/662) had also accessed other forms of support, although 63.2% (410/649) reported wanting additional support. Both use and perceived need for additional support were concentrated amongst minoritised and more psychosocially vulnerable groups.

## AI/LLM platforms as an emerging source of adolescent mental health support

General-purpose generative artificial intelligence (AI) and large language model (LLM) chatbots are rapidly emerging as a resource for mental health support^1–3^, yet research and regulation struggle to keep pace with rapid technological advancements and shifts in how individuals are using these tools^4,5^. At present, there is little clarity regarding what direct clinical input or supervision, if any, has been incorporated into the development and deployment of these platforms, and whether the present safety guardrails and crisis response features are clinically appropriate, aligned with safeguarding frameworks, or tailored to users’ needs^6,7^. Regulatory frameworks to enable monitoring of these platforms—including both potential risks and benefits—are in their nascent stages^4,8,9^, generating a landscape of ‘uncertainty, risk, and potential’^6^.

Adolescents represent a particularly important group in this context. Adolescence, broadly encompassing ages 10–24 years^10^, has long been recognised as a uniquely important developmental stage in terms of mental health^11^, with today’s adolescents experiencing earlier onset alongside increases in the prevalence, severity, and impact of mental health difficulties^12–16^. For those seeking support, AI/LLM platforms may offer the present generation of ‘digital natives’ an alternative or complement to more traditional forms of support, particularly where individual-, family- or service-level barriers make other avenues of support less available, accessible, or acceptable^17,18^.

Estimates of how many adolescents are using AI/LLMs for mental health support vary. The proliferation of these platforms, technological advances in their capabilities, and lack of agreed-upon definitions as to what types of engagement constitute ‘mental health support’ all complicate assessment of usage patterns^5^. Recent estimates from Europe^18,19^ and the United States^1,2^ suggest that as many as one in four young people might be using these tools for mental health advice and/or support, meaning they are relatively common when compared with other forms of support^20,21^. There is also emerging evidence of these tools’ acceptability, with American studies estimating that around nine in ten young people who had used AI/LLMs for mental health advice viewed the support received as ‘somewhat’ or ‘very’ helpful^1,2^.

Where AI/LLMs sit within adolescents’ ecosystem of support—be it as adaptive digital help-seeking, a stopgap response to unmet clinical need, or even a potentially harmful substitute for clinical care—remains underexplored. As countries around the world navigate a pivotal moment in both digital regulation and youth mental health, a better understanding of how adolescents use these platforms in the context of mental health support is essential for informing policy and practice^22–24^.

To contextualise how these rapidly-evolving platforms fit within today’s adolescents’ networks of care^21^, we conducted a series of exploratory analyses of data from a large, school-based survey in England to address four research questions:

RQ1. How common is use of AI/LLM platforms for mental health support amongst adolescents, and who is most likely to use it?

RQ2. How helpful do adolescents find AI/LLM platforms for mental health support, and who is most likely to find them helpful?

RQ3. What is the degree of perceived unmet need amongst adolescents who use AI/LLM platforms for mental health support, and who is most likely to report unmet need?

RQ4. Are adolescents using AI/LLM platforms as a substitute for or supplement to other types of mental health support?

## Adolescents’ use and experiences of AI/LLMs as a source of mental health support

**Fig. 1:**
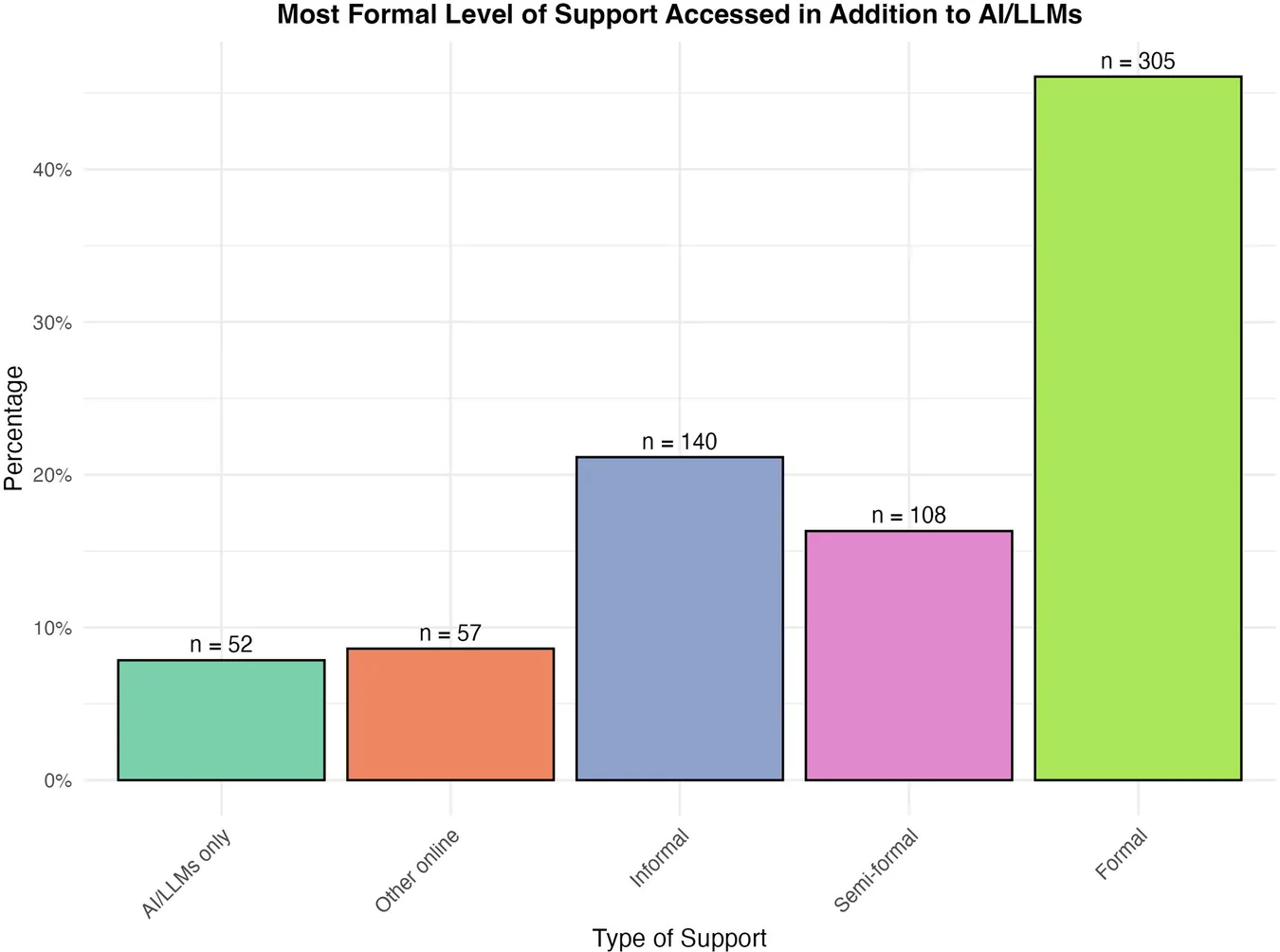
Most formal level of mental health support accessed in addition to AI/LLM platforms (*N* = 662). See Tables S4 and S5 for detail on what types of support are included in each category.

In our school-based sample of 24,132 adolescents, 662 (2.7%) reported using AI/LLM platforms for mental health support in the last year (RQ1). Of these, 55.9% (324/580) rated support received as ‘helpful’, 31.4% (182/580) as ‘neither helpful nor unhelpful’, and 12.8% (74/580) as ‘unhelpful’ (RQ2; Fig. S1). Amongst those who had used AI/LLM platforms for support, 63.2% (410/649) reported wanting additional support (RQ3; Fig. S2). Notably, most adolescents who used AI/LLM platforms had also accessed other types of mental health support (92.1%; 610/662), with nearly half (46.1%; 305/662) reporting having accessed formal services in the previous year (Fig. 1; RQ4).

**Fig. 2:**
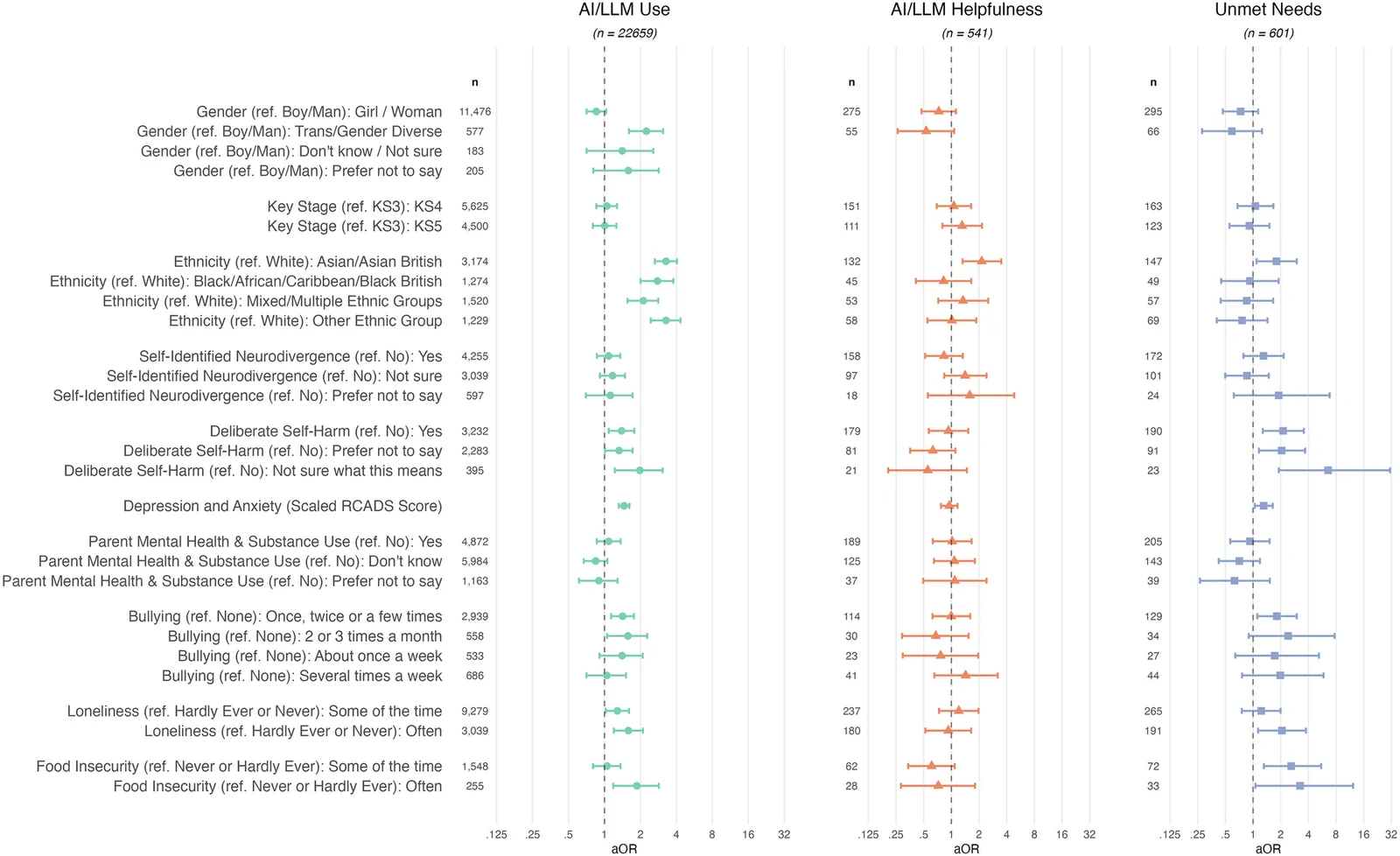
Adjusted odds ratios from the regression analyses of the reported use and helpfulness of AI/LLM platforms for mental health support as well as perceived unmet need in the context of having accessed AI/LLM platforms. N_use_ = 22,659; N_helpfulness_ = 541; N_unmet needs_ = 601. Notes. Two gender categories are not plotted for helpfulness and unmet need due to disclosive cell counts (<5). Full results from the regression analysis are presented in Table S1-S3. KS3, KS4 and KS5 refer to stages of the English educational system: Key Stage 3 (~11–14 years), Key Stage 4 (~14–16 years) and Key Stage 5 (~16–18 years).

In the regression analyses (Fig. 2), adolescents were significantly more likely to have used AI/LLM platforms (RQ1) if they were trans or gender diverse (aOR: 2.24, 95% CI: 1.61–3.09); had experienced food insecurity (aOR for *‘often’* experiencing food insecurity: 1.87, 95% CI: 1.18–2.85); or were from any minoritised ethnic background, with the largest effects for those from Asian/Asian British (aOR 3.27, 95% CI: 2.64–4.04) and Other (aOR 3.27, 95% CI: 2.44–4.33) ethnic groups. Adolescents who had elevated depression/anxiety (aOR per 1 SD increase on the RCADS-25: 1.46, 95% CI: 1.32–1.62), had ever self-harmed (aOR: 1.39, 95% CI: 1.09–1.78), or reported loneliness (aOR for *‘often’* lonely: 1.58, 95% CI: 1.20–2.10) were also more likely to report using these platforms for mental health support. Experience of bullying was also associated with increased likelihood of accessing AI/LLM platforms for support, but only at more infrequent levels of bullying (aORs for being bullied *‘once, twice or a few times’* in recent months: 1.42, 95% CI: 1.14–1.76 and for *'2 or 3 times a month'*: 1.57, 95% CI: 1.05–2.28).

In contrast, there were generally no differences across groups regarding perceived helpfulness of AI/LLM support (RQ2). The only group that was significantly more likely to find AI/LLM platforms helpful were Asian/Asian British adolescents (aOR: 2.14, 95% CI 1.33–3.50).

Finally, adolescents were significantly more likely to report wanting additional support (i.e. to have perceived unmet need; RQ3) if they were Asian/Asian British (aOR: 1.80, 95% CI: 1.09–2.99), had elevated depression/anxiety (aOR per 1 SD increase on the RCADS-25: 1.30, 95% CI: 1.05–1.64), had ever self-harmed (aOR: 2.12, 95% CI: 1.28–3.57), or felt lonely ‘often’ (aOR: 2.06, 95% CI: 1.13–3.77). Low-frequency bullying (aOR for *‘once, twice, or a few times’*: 1.81, 95% CI: 1.11–2.99) and food insecurity (aORs for *‘some of the time’*: 2.60, 95% CI: 1.31, 5.51 and for* 'often':* 3.25, 95% CI: 1.06–12.28) were also each associated with increased likelihood of perceived unmet need.

Tables S1–S3 present full descriptives and regression results.

## Contextualisation within a rapidly evolving evidence base

Use of general-purpose AI/LLM platforms for mental health support was reported by a relatively small proportion (2.7%) of adolescents in our large 2025 school-based survey in England, with higher use observed amongst minoritised and more psychosocially vulnerable groups. Most adolescents (92.1%) who had used AI/LLM platforms had also sought help elsewhere, suggesting that these platforms are a *supplement* to, rather than a *substitute* for, other forms of support. Whilst just over half (55.9%) of participants who had used AI/LLMs for mental health support rated them as ‘helpful’, around two-thirds (63.2%) reported wanting additional support, suggesting that AI/LLM these platforms are potentially *filling* a gap without *closing* it.

These findings add to a growing evidence base about the role of AI/LLMs in mental health support. Whilst our estimate of the proportion of adolescents using these tools for support is lower than others e.g., 1, 2, 18, 19, direct comparison must also be approached with caution, as differences in study methodology—including how ‘mental health support’ is defined and operationalised—have created substantial heterogeneity^5^. Many studies have been framed around *how individuals are using AI/LLMs*, with mental health support presented alongside other uses. Conversely, the OxWell survey was framed around *access to mental health support*, with AI/LLMs presented alongside support options ranging from informational websites through to specialist mental health services. This approach may have resulted in a more conservative estimate.

It is also important to note that this is an area in flux—as AI/LLMs become more capable, readily accessible, and increasingly integrated into everyday digital platforms, their role in mental health support will also evolve^5^. McBain and colleagues’ work provides a useful illustration, with an approximate 50% increase in AI chatbot use for mental health advice between their February–March 2025 (13.1%^2^) and November 2025 (19.2%^1^) survey administrations. Our findings should therefore be viewed within their temporal and technological context. It is reasonable to suggest that the proportion of adolescents using these platforms will have increased since the 2025 survey, and that modes of engagement will have evolved alongside the platforms themselves.

Beyond providing an estimate of how many adolescents might be using AI/LLMs  for mental health support, our findings add significant nuance toward understanding adolescents’ experiences of these platforms at scale. That over half of adolescents perceived AI/LLMs as ‘helpful’ aligns with other quantitative^1,2^ and qualitative^17^ evidence, and suggests that there may be a place for these tools within the broader youth mental health landscape. However, we must not ignore the one-in-eight adolescents who reported that AI/LLMs were ‘unhelpful’, particularly in light of potential risks^25^. It is also worth noting that, whilst many found these tools *helpful*, they were often not seen as *sufficient*, as suggested by the fact that two-thirds of AI/LLM users indicated that they wanted additional support. This underscores how AI/LLM platforms and more traditional forms of support do not exist in isolation—a notion also reflected in the fact that the vast majority of those who sought support from AI/LLMs had also received support from friends and family, school and community, and/or formal services. The remainder (16.5%) used only online sources for mental health support, around half of whom used *only* AI/LLM support. These adolescents are a clinically relevant group who may be less visible to conventional services and therefore harder to identify and support.

The evidence base around the role of AI/LLMs in mental health support is nascent and evolving^3,5^, and whilst our findings help elucidate adolescents’ use and experiences of these tools, perceived helpfulness captures only one dimension of the quality and potential impact of this support. Rigorous and systematic evaluation of the potential benefits and harms of these platforms must therefore be a priority for empirical research^26^. These early findings do, however, provide valuable, real-world insight into an emerging area at the intersection of adolescent mental health and digital landscapes. Clinicians, schools, and policymakers should be aware that those engaging with these platforms are likely a more vulnerable group who may benefit from additional support. These findings would also suggest that, for many adolescents, these tools can also be a helpful complement to existing support and should therefore be acknowledged and explored within wider conversations about how the current generation of digital natives seeks and experiences mental health support.

## Methods

### Data source

This study used data from the secondary school version of the 2025 wave of the OxWell Student Survey^27^, a repeated cross-sectional survey of the mental health and wellbeing of students in English secondary schools and further education colleges (FECs)—hereafter ‘schools’. Sampling was non-probabilistic: school-level recruitment occurred primarily via local authority partners, which invited all schools in their catchment area to participate, as well as through a multi-academy trust and an FEC group which had at least one school within these local authority catchment areas. Participating schools enrolled students using (1) a parental opt-out model and student assent (student age <16 years) or (2) students’ informed consent only (student age ≥16 years).

The survey was administered during the school day and did not collect identifiable data about students to increase the diversity and representativeness of the sample, enable a parental opt-out model to ensure maximum participation, and encourage more accurate and honest responses^27,28^. Responses for this wave were collected February–May 2025 in schools primarily located in Oxfordshire and Liverpool, England. A complete description of the 2025 OxWell wave is available on the OSF (https://osf.io/sekhr/files/m59jh)^29^.

Ethical approval for the 2025 wave was granted by the University of Oxford Medical Sciences Interdivisional Research Ethics Committee (reference: R62366/RE0017).

### Participants

This analysis focused on adolescents in Years 7 to 13 (primary age range 11–18 years) at participating secondary schools and FECs (FEC participants, whose ages can extend up to ~21 years, were assigned to derived year groups based on academic level to ensure comparability with the secondary school cohort). In total, 36,169 students logged into the online survey platform. We excluded 103 participants from four institutions (two alternative provision/non-mainstream schools and two with minimal/irregular engagement) and a further 3964 students who did not consent, for a total of 32,102 participants (89% consent rate for those who logged into the platform). Of the 32,102 total potential participants, 24,132 (75.2%) reached the survey section pertaining to whether they had accessed AI/LLMs for mental health support and were therefore eligible for inclusion in the present analysis.

In the final analytical sample, 50.7% were girls, 44.7% boys, and 2.7% trans/gender diverse, with a further 0.8% indicating they were unsure of their gender identity and 1.0% who did not wish to disclose their gender. Excluding 317 participants (1.3%) who were >18 years (whose precise age we could not determine due to response option framing, i.e. ‘19 + ’ for secondary schools and ‘21 + ’ for FECs), the mean age was 14.0 years (SD = 1.9). In terms of key stage, 55.7% of participants were in Key Stage 3 (ages 11–14), 24.7% in Key Stage 4 (ages 14–16), and 19.6% in Key Stage 5 (ages 16–18). Participants mainly identified as White (68.0%), followed by 14.0% as Asian, 6.7% as mixed/multiple ethnic groups, 5.8% as Black, and 5.5% as ‘other’ ethnic group. Full descriptive statistics are provided in Table S1.

### Measures

Precise question wording, response options, and data processing descriptions are provided in Tables S4 and S5, and the full 2025 OxWell variable guide is available on the OSF (https://osf.io/r6tnm).

### Outcomes

Past-year use of AI/LLMs for mental health support (RQ1) was assessed according to whether students endorsed having accessed ‘AI/LLM platforms (e.g. ChatGPT, Gemini, Copilot)’ for mental health support in the past year.

For those who reported having accessed AI/LLMs for support, the perceived helpfulness of this support (RQ2) was assessed through the question ‘*How helpful was the support?’* (response options: ‘Unhelpful’—‘Neither helpful nor unhelpful’—‘Helpful’). For descriptive analyses, this outcome was reported according to the three response categories, whilst for regression analyses, responses were collapsed into ‘helpful’ (comprising ‘helpful’ only) and ‘not helpful’ (comprising ‘unhelpful’ and ‘neither helpful nor unhelpful’) to aid interpretability.

Perceived unmet needs for mental health support amongst those who had used AI/LLMs (RQ3) were assessed according to whether students endorsed any option other than ‘No, I did not want additional support’ for the question *‘In the last year, would you have liked to access additional mental health support from any of the following…*[types of support]*’*.

Finally, for those who reported having used AI/LLMs for mental health support, whether this was as a *substitute for* or *supplement to* other forms of mental health support (RQ4) was assessed according to whether students reported having accessed any other type of mental health support across any other domain in the past year. For this outcome, we created a new variable with five categories coded according to the ‘most formal’ level of support accessed in addition to AI/LLMs as follows:

- *Formal:* AI/LLMs + support from health services/social care sources ± semiformal, informal, or other online support
- *Semiformal:* AI/LLMs + support from school/community sources ± informal or other online support
- *Informal:* AI/LLMs + support from informal sources ± other online support
- *Other online:* AI/LLMs + other online support
- *AI/LLMs only* (no other support accessed)

Table S4 provides full details of the outcome variables.

### Predictors

We examined our four outcomes across ten different variables grouped into three conceptual domains: identity factors, mental health and self-harm, and social adversity. Identity factors included gender (assessed using one question on gender identity and one on trans identity), key stage (representing year groupings in the English education system), ethnicity (assessed according to categories used by the Office for National Statistics), and self-identified neurodivergence. Mental health and self-harm included depression and anxiety symptoms (assessed using the 25-item version of the Revised Children’s Anxiety and Depression Scale (RCADS-25^30^)) and lifetime self-harm. Social adversity included bullying, loneliness, food insecurity, and a composite measure of parental mental health and parental substance use. Table S5 provides full details of the predictor variables.

### Analysis

To provide an overview of how AI/LLMs are being used for mental health support, we first report findings for all four outcomes (RQ1-4) descriptively (*n (%)* or *mean (standard deviation)*, as relevant). For outcomes pertaining to AI/LLM use, perceived helpfulness, and perceived unmet need (RQ1-3), we additionally examined associations through bivariate analyses (yielding unadjusted odds ratios (ORs)) and complete-case logistic regression using the predictors outlined above (yielding adjusted ORs (aORs)) in order to examine variation across student characteristics and experiences. We did not conduct a regression analysis for the substitution vs. supplementation outcome (RQ4) due to the very low number of students (*n* = 52) reporting *only* using AI/LLMs for support. We conducted all analyses in R version 4.2.3 using Release 14 of the OxWell 2025 data. The statistical analysis code is available on GitHub (https://github.com/OxWellStudy/AI-MH-Support/).

## Supplementary information

Supplementary information

## Data Availability

Fully de-identified extracts of the data can be provided to academic research collaborators upon reasonable request, following a careful review process by the research team. The data are not publicly available due to ethical and information governance restrictions.

## Code availability

The underlying code for this study is available in GitHub and can be accessed via this link: https://github.com/OxWellStudy/AI-MH-Support/.
