# Supplementary material for: General-purpose AI as a source of mental health support amongst 24,000 adolescents in England
Source: Npj Ment Health Res. 2026 Oct 27;5:55. doi: 10.1038/s44184-026-00254-x (PMC13754904; doi:10.1038/s44184-026-00254-x)
Supplement: Supplementary file 1 [file 44184_2026_254_MOESM1_ESM.pdf]

Table S1 | Sample descriptives by past-year use of AI/LLMs for mental health support (RQ1)

	Full sample (n = 24,132)	No AI/LLM Use (n = 23,470)	AI/LLM Use (n = 662)	Unadj. OR [95% CI]	p	Adj. OR [95% CI]	p
**Identity factors**							
**Gender** *n (%)*							
Boy/Man	10770 (44.7)	10526 (97.7)	244 (2.27)	Ref.	Ref.	Ref.	Ref.
Girl/Woman	12209 (50.7)	11894 (97.4)	315 (2.58)	1.14 [0.96, 1.35]	0.123	0.86 [0.71, 1.03]	0.105
Trans/Gender Diverse	650 (2.7)	575 (88.5)	75 (11.5)	5.63 [4.27, 7.36]	<0.001	2.24 [1.61, 3.09]	<0.001
Don't know/Not sure	203 (0.8)	190 (93.6)	13 (6.40)	2.98 [1.59, 5.11]	0.001	1.41 [0.71, 2.56]	0.311
Prefer not to say	251 (1.0)	238 (94.8)	13 (5.18)	2.38 [1.28, 4.06]	0.008	1.58 [0.81, 2.85]	0.172
**Key Stage** *n (%)*							
KS3	13434 (55.7)	13089 (97.4)	345 (2.57)	Ref.	Ref.	Ref.	Ref.
KS4	5969 (24.7)	5788 (97.0)	181 (3.03)	1.19 [0.99, 1.42]	0.068	1.04 [0.85, 1.27]	0.665
KS5	4729 (19.6)	4593 (97.1)	136 (2.88)	1.12 [0.92, 1.37]	0.258	1.01 [0.80, 1.26]	0.962
**Ethnicity** *n (%)*							
White	16081 (68.0)	15783 (98.1)	298 (1.85)	Ref.	Ref.	Ref.	Ref.
Asian/Asian British	3303 (14.0)	3145 (95.2)	158 (4.78)	2.66 [2.18, 3.24]	<0.001	3.27 [2.64, 4.04]	<0.001
Black/African/Caribbean/Black British	1369 (5.8)	1313 (95.9)	56 (4.09)	2.26 [1.68, 3.00]	<0.001	2.77 [2.00, 3.77]	<0.001
Mixed/Multiple Ethnic Groups	1576 (6.7)	1518 (96.3)	58 (3.68)	2.03 [1.51, 2.68]	<0.001	2.11 [1.56, 2.82]	<0.001
Other Ethnic Group	1304 (5.5)	1234 (94.6)	70 (5.37)	3.01 [2.29, 3.91]	<0.001	3.27 [2.44, 4.33]	<0.001
**Self-Identified Neurodivergence** *n (%)*							
No	15444 (64.7)	15115 (97.9)	329 (2.13)	Ref.	Ref.	Ref.	Ref.
Yes	4497 (18.8)	4309 (95.8)	188 (4.18)	2.01 [1.67, 2.40]	<0.001	1.08 [0.86, 1.35]	0.489
Not sure	3252 (13.6)	3142 (96.6)	110 (3.38)	1.61 [1.29, 2.00]	<0.001	1.17 [0.92, 1.48]	0.208
Prefer not to say	673 (2.8)	646 (96.0)	27 (4.01)	1.93 [1.26, 2.83]	0.003	1.12 [0.70, 1.72]	0.623
**Mental health & self-harm**							
**Deliberate Self-Harm** *n (%)*							
No	17462 (73.7)	17134 (98.1)	328 (1.88)	Ref.	Ref.	Ref.	Ref.
Yes	3364 (14.2)	3164 (94.1)	200 (5.95)	3.30 [2.76, 3.95]	<0.001	1.39 [1.09, 1.78]	0.009
Prefer not to say	2430 (10.3)	2332 (96.0)	98 (4.03)	2.20 [1.74, 2.75]	<0.001	1.32 [1.01, 1.72]	0.041

Not sure what this means	445 (1.9)	418 (93.9)	27 (6.07)	3.39 [2.21, 4.99]	<0.001	1.98 [1.22, 3.06]	0.007
**Depression & Anxiety (RCADS Score)** *mean (SD)*	50.5 (16.8)	50.2 (16.5)	63.1 (20.5)	-	-	-	-
**Depression & Anxiety (Scaled RCADS Score)**	-	-	-	1.83 [1.72, 1.95]	<0.001	1.46 [1.32, 1.62]	<0.001
**Social adversity**							
**Parental Mental Health and Substance Use Problems** *n (%)*							
No	11203 (46.7)	10968 (97.9)	235 (2.10)	Ref.	Ref.	Ref.	Ref.
Yes	5126 (21.4)	4906 (95.7)	220 (4.29)	2.09 [1.74, 2.52]	<0.001	1.08 [0.86, 1.37]	0.490
Don't know	6373 (26.6)	6217 (97.6)	156 (2.45)	1.17 [0.95, 1.44]	0.132	0.84 [0.67, 1.06]	0.141
Prefer not to say	1279 (5.3)	1234 (96.5)	45 (3.52)	1.71 [1.22, 2.34]	0.002	0.90 [0.61, 1.29]	0.566
**Bullying** *n (%)*							
Nil in past 2 months	18875 (78.9)	18473 (97.9)	402 (2.13)	Ref.	Ref.	Ref.	Ref.
Once, twice or a few times	3108 (13.0)	2972 (95.6)	136 (4.38)	2.10 [1.72, 2.56]	<0.001	1.42 [1.14, 1.76]	0.002
2 or 3 times a month	595 (2.5)	558 (93.8)	37 (6.22)	3.06 [2.13, 4.27]	<0.001	1.57 [1.05, 2.28]	0.028
About once a week	585 (2.5)	557 (95.2)	28 (4.79)	2.32 [1.53, 3.37]	<0.001	1.40 [0.91, 2.09]	0.123
Several times a week	748 (3.1)	695 (92.9)	53 (7.09)	3.51 [2.58, 4.68]	<0.001	1.05 [0.71, 1.51]	0.817
**Loneliness** *n (%)*							
Hardly ever or never	10872 (45.4)	10713 (98.5)	159 (1.46)	Ref.	Ref.	Ref.	Ref.
Some of the time	9873 (41.2)	9582 (97.1)	291 (2.95)	2.05 [1.69, 2.49]	<0.001	1.28 [1.02, 1.61]	0.030
Often	3214 (13.4)	3009 (93.6)	205 (6.38)	4.59 [3.72, 5.67]	<0.001	1.58 [1.20, 2.10]	0.001
**Food Insecurity** *n (%)*							
Never or hardly ever	22047 (91.9)	21506 (97.5)	541 (2.45)	Ref.	Ref.	Ref.	Ref.
Some of the time	1656 (6.9)	1578 (95.3)	78 (4.71)	1.97 [1.53, 2.49]	<0.001	1.05 [0.80, 1.36]	0.711
Often	296 (1.2)	258 (87.2)	38 (12.8)	5.87 [4.07, 8.24]	<0.001	1.87 [1.18, 2.85]	0.008

Notes. aORs estimated from logistic model using complete cases (n = 22,659). KS3, KS4 and KS5 refer to stages of the English educational system: Key Stage 3 (approximately 11-14 years), Key Stage 4 (approximately 14-16 years) and Key Stage 5 (approximately 16-18 years).

Table S2 | Sample descriptives by reported helpfulness of AI/LLMs for mental health support (RQ2)

	AI/LLM Support Unhelpful (n = 74)	AI/LLM Support Neither Helpful nor Unhelpful (n = 182)	AI/LLM Support Helpful (n = 324)	Unadj. OR (ref. = unhelpful/neither) [95% CI]	p	Adj. OR (ref. = unhelpful/neither) [95% CI]	p
**Identity factors**							
**Gender** *n (%)*							
Boy/Man	24 (11.7)	59 (28.6)	123 (59.7)	Ref.	Ref.	Ref.	Ref.
Girl/Woman	28 (9.62)	99 (34.0)	164 (56.4)	0.87 [0.61, 1.25]	0.458	0.73 [0.47, 1.12]	0.151
Trans/Gender Diverse	15 (24.6)	20 (32.8)	26 (42.6)	0.50 [0.28, 0.90]	0.020	0.53 [0.26, 1.07]	0.078
Don't know/Not sure	N.R.	N.R.	N.R.	N.R.	N.R.	N.R.	N.R.
Prefer not to say	N.R.	N.R.	N.R.	N.R.	N.R.	N.R.	N.R.
**Key Stage** *n (%)*							
KS3	42 (14.0)	93 (31.1)	164 (54.8)	Ref.	Ref.	Ref.	Ref.
KS4	22 (13.6)	54 (33.3)	86 (53.1)	0.93 [0.63, 1.37]	0.718	1.07 [0.69, 1.64]	0.774
KS5	10 (8.40)	35 (29.4)	74 (62.2)	1.35 [0.88, 2.10]	0.174	1.31 [0.80, 2.16]	0.289
**Ethnicity** *n (%)*							
White	35 (13.3)	90 (34.2)	138 (52.5)	Ref.	Ref.	Ref.	Ref.
Asian/Asian British	8 (5.71)	36 (25.7)	96 (68.6)	1.97 [1.28, 3.05]	0.002	2.14 [1.33, 3.50]	0.002
Black/African/Caribbean/Black British	10 (20.8)	15 (31.2)	23 (47.9)	0.83 [0.45, 1.55]	0.566	0.82 [0.41, 1.65]	0.580
Mixed/Multiple Ethnic Groups	5 (9.43)	18 (34.0)	30 (56.6)	1.18 [0.65, 2.16]	0.588	1.34 [0.72, 2.52]	0.358
Other Ethnic Group	14 (24.1)	15 (25.9)	29 (50.0)	0.91 [0.51, 1.61]	0.735	1.01 [0.55, 1.87]	0.970
**Self-Identified Neurodivergence** *n (%)*							
No	24 (8.42)	90 (31.6)	171 (60.0)	Ref.	Ref.	Ref.	Ref.
Yes	39 (23.1)	51 (30.2)	79 (46.7)	0.59 [0.40, 0.86]	0.006	0.83 [0.52, 1.33]	0.438
Not sure	9 (8.65)	33 (31.7)	62 (59.6)	0.98 [0.62, 1.56]	0.943	1.41 [0.84, 2.41]	0.197
Prefer not to say	N.R.	N.R.	11 (57.9)	0.91 [0.35, 2.46]	0.851	1.59 [0.55, 4.84]	0.392
**Mental health & self-harm**							
**Deliberate Self-Harm** *n (%)*							

No	24 (8.57)	83 (29.6)	173 (61.8)	Ref.	Ref.	Ref.	Ref.
Yes	34 (18.4)	54 (29.2)	97 (52.4)	0.68 [0.47, 0.99]	0.047	0.93 [0.57, 1.53]	0.773
Prefer not to say	13 (15.1)	32 (37.2)	41 (47.7)	0.56 [0.35, 0.92]	0.022	0.63 [0.36, 1.11]	0.107
Not sure what this means	N.R.	N.R.	12 (50.0)	0.62 [0.26, 1.46]	0.268	0.55 [0.21, 1.48]	0.235
**Depression & Anxiety (RCADS Score)** *mean (SD)*	71.7 (25.5)	64.2 (17.7)	62.1 (19.6)	-	-		
**Depression & Anxiety (Scaled RCADS Score)**	-	-	-	0.83 [0.73, 0.96]	0.011	0.95 [0.77, 1.17]	0.615
**Social adversity**							
**Parental Mental Health and Substance Use Problems** *n (%)*							
No	23 (11.2)	65 (31.7)	117 (57.1)	Ref.	Ref.	Ref.	Ref.
Yes	32 (16.2)	59 (29.9)	106 (53.8)	0.88 [0.59, 1.30]	0.512	1.02 [0.63, 1.66]	0.939
Don't know	13 (9.70)	44 (32.8)	77 (57.5)	1.02 [0.65, 1.58]	0.945	1.08 [0.65, 1.80]	0.776
Prefer not to say	6 (14.6)	13 (31.7)	22 (53.7)	0.87 [0.44, 1.73]	0.689	1.09 [0.49, 2.41]	0.833
**Bullying** *n (%)*							
Nil in past 2 months	36 (10.0)	113 (31.5)	210 (58.5)	Ref.	Ref.	Ref.	Ref.
Once, twice or a few times	18 (15.5)	35 (30.2)	63 (54.3)	0.84 [0.55, 1.29]	0.430	1.00 [0.62, 1.60]	0.993
2 or 3 times a month	6 (18.8)	13 (40.6)	13 (40.6)	0.49 [0.23, 1.02]	0.055	0.68 [0.29, 1.54]	0.351
About once a week	N.R.	N.R.	11 (47.8)	0.65 [0.27, 1.54]	0.326	0.76 [0.30, 1.96]	0.573
Several times a week	11 (23.4)	12 (25.5)	24 (51.1)	0.74 [0.40, 1.37]	0.338	1.43 [0.65, 3.20]	0.372
**Loneliness** *n (%)*							
Hardly ever or never	13 (9.63)	45 (33.3)	77 (57.0)	Ref.	Ref.	Ref.	Ref.
Some of the time	21 (8.33)	84 (33.3)	147 (58.3)	1.05 [0.69, 1.61]	0.805	1.20 [0.73, 1.98]	0.465
Often	40 (21.3)	53 (28.2)	95 (50.5)	0.77 [0.49, 1.20]	0.251	0.92 [0.52, 1.64]	0.789
**Food Insecurity** *n (%)*							
Never or hardly ever	55 (11.4)	144 (29.9)	282 (58.6)	Ref.	Ref.	Ref.	Ref.
Some of the time	9 (14.1)	27 (42.2)	28 (43.8)	0.55 [0.32, 0.93]	0.026	0.61 [0.34, 1.09]	0.095
Often	9 (28.1)	11 (34.4)	12 (37.5)	0.43 [0.20, 0.89]	0.022	0.72 [0.28, 1.81]	0.487

Notes. aORs estimated from logistic model using complete cases (n = 541). Values <5 are marked as 'N.R.' to avoid risk of disclosure. KS3, KS4 and KS5 refer to stages of the English educational system: Key Stage 3 (approximately 11-14 years), Key Stage 4 (approximately 14-16 years) and Key Stage 5 (approximately 16-18 years).

Table S3 | Sample descriptives by perceived unmet needs in the context of AI/LLM use for mental health support (RQ3)

	No Perceived Unmet Need (n = 239)	Perceived Unmet Need (n =410)	Unadj. OR [95% CI]	p	Adj. OR [95% CI]	p
**Identity factors**						
**Gender** *n (%)*						
Boy/Man	96 (40.5)	141 (59.5)	Ref.	Ref.	Ref.	Ref.
Girl/Woman	118 (38.1)	192 (61.9)	1.11 [0.78, 1.57]	0.563	0.73 [0.47, 1.13]	0.164
Trans/Gender Diverse	20 (26.7)	55 (73.3)	1.86 [1.06, 3.37]	0.030	0.59 [0.28, 1.25]	0.166
Don't know/Not sure	N.R.	N.R.	N.R.	N.R.	N.R.	N.R.
Prefer not to say	N.R.	N.R.	N.R.	N.R.	N.R.	N.R.
**Key Stage** *n (%)*						
KS3	119 (35.2)	219 (64.8)	Ref.	Ref.	Ref.	Ref.
KS4	65 (36.3)	114 (63.7)	0.95 [0.65, 1.39]	0.802	1.06 [0.68, 1.66]	0.805
KS5	55 (41.7)	77 (58.3)	0.76 [0.50, 1.15]	0.196	0.91 [0.55, 1.51]	0.720
**Ethnicity** *n (%)*						
White	110 (37.8)	181 (62.2)	Ref.	Ref.	Ref.	Ref.
Asian/Asian British	45 (29.0)	110 (71.0)	1.48 [0.98, 2.27]	0.064	1.80 [1.09, 2.99]	0.021
Black/African/Caribbean/Black British	25 (47.2)	28 (52.8)	0.68 [0.38, 1.24]	0.205	0.92 [0.45, 1.90]	0.828
Mixed/Multiple Ethnic Groups	22 (37.9)	36 (62.1)	0.99 [0.56, 1.80]	0.979	0.85 [0.44, 1.66]	0.635
Other Ethnic Group	29 (41.4)	41 (58.6)	0.86 [0.51, 1.47]	0.576	0.76 [0.40, 1.44]	0.396
**Self-Identified Neurodivergence** *n (%)*						
No	142 (44.0)	181 (56.0)	Ref.	Ref.	Ref.	Ref.
Yes	49 (26.3)	137 (73.7)	2.19 [1.48, 3.26]	<0.001	1.30 [0.78, 2.15]	0.309
Not sure	40 (37.0)	68 (63.0)	1.33 [0.85, 2.10]	0.209	0.86 [0.50, 1.48]	0.582
Prefer not to say	6 (23.1)	20 (76.9)	2.56 [1.05, 7.27]	0.038	1.90 [0.62, 6.82]	0.273
**Mental health & self-harm**						
**Deliberate Self-Harm** *n (%)*						
No	158 (49.2)	163 (50.8)	Ref.	Ref.	Ref.	Ref.
Yes	45 (22.6)	154 (77.4)	3.30 [2.23, 4.96]	<0.001	2.12 [1.28, 3.57]	0.004

Prefer not to say	29 (29.9)	68 (70.1)	2.26 [1.40, 3.73]	0.001	2.05 [1.16, 3.69]	0.013
Not sure what this means	5 (19.2)	21 (80.8)	3.96 [1.56, 12.3]	0.003	6.56 [1.90, 31.0]	0.002
**Depression & Anxiety (RCADS Score)** *mean (SD)*	56.0 (17.4)	67.7 (20.9)	-	-		
**Depression & Anxiety (Scaled RCADS Score)**	-	-	1.70 [1.46, 1.99]	<0.001	1.30 [1.05, 1.64]	0.018
**Social adversity**						
**Parental Mental Health and Substance Use Problems** *n (%)*						
No	100 (43.1)	132 (56.9)	Ref.	Ref.	Ref.	Ref.
Yes	66 (30.6)	150 (69.4)	1.72 [1.17, 2.54]	0.006	0.93 [0.56, 1.51]	0.757
Don't know	57 (37.5)	95 (62.5)	1.26 [0.83, 1.92]	0.277	0.71 [0.42, 1.19]	0.192
Prefer not to say	14 (32.6)	29 (67.4)	1.56 [0.79, 3.20]	0.202	0.63 [0.26, 1.52]	0.299
**Bullying** *n (%)*						
Nil in past 2 months	180 (45.8)	213 (54.2)	Ref.	Ref.	Ref.	Ref.
Once, twice or a few times	37 (27.4)	98 (72.6)	2.23 [1.46, 3.45]	<0.001	1.81 [1.11, 2.99]	0.017
2 or 3 times a month	6 (16.2)	31 (83.8)	4.27 [1.85, 11.7]	<0.001	2.41 [0.90, 7.70]	0.083
About once a week	6 (22.2)	21 (77.8)	2.90 [1.20, 8.15]	0.016	1.72 [0.64, 5.21]	0.291
Several times a week	8 (15.4)	44 (84.6)	4.56 [2.20, 10.8]	<0.001	1.98 [0.76, 5.85]	0.169
**Loneliness** *n (%)*						
Hardly ever or never	78 (50.0)	78 (50.0)	Ref.	Ref.	Ref.	Ref.
Some of the time	114 (40.1)	170 (59.9)	1.49 [1.00, 2.21]	0.047	1.23 [0.75, 2.00]	0.410
Often	46 (22.7)	157 (77.3)	3.40 [2.16, 5.39]	<0.001	2.06 [1.13, 3.77]	0.018
**Food Insecurity** *n (%)*						
Never or hardly ever	219 (41.3)	311 (58.7)	Ref.	Ref.	Ref.	Ref.
Some of the time	13 (17.1)	63 (82.9)	3.38 [1.87, 6.58]	<0.001	2.60 [1.31, 5.51]	0.006
Often	5 (13.2)	33 (86.8)	4.52 [1.89, 13.6]	<0.001	3.25 [1.06, 12.3]	0.039

Notes. aORs estimated from logistic model using complete cases (n = 601). Values <5 are marked as 'N.R.' to avoid risk of disclosure. KS3, KS4 and KS5 refer to stages of the English educational system: Key Stage 3 (approximately 11-14 years), Key Stage 4 (approximately 14-16 years) and Key Stage 5 (approximately 16-18 years).

Table S4 | Outcome variables used in the analyses

**Outcome variable**	**Question wording**	**Response options**	**Answer type**	**Additional processing (if any)**	**Analysis notes (if any)**
**Past-year use of AI/LLMs** for mental health support (RQ1)	In the last year, have you received mental health support from any of the following **digital and online sources,** that you haven't already told us about?<br><br>*Please tick all that apply. Leave blank if not received.*	• Helplines (telephone, text, app, or web based - e.g. Childline, Kooth, TellMi, BetterHelp)<br>• Official informational websites or apps (e.g. NHS websites, ChatHealth)<br>• Other web-based information platforms (e.g. YouTube, blogs)<br>• Social media posts (e.g. TikTok, Instagram)<br>• Online communities (e.g. chatrooms, forums)<br>• **AI/LLM platforms (e.g. ChatGPT, Gemini, Copilot)**<br>• Other	Tickbox	If AI/LLMs were selected, recoded as 'Yes'. If tickbox left blank, recoded as 'No'*<br><br>**As we restricted our sample to those who reached this question, non-response within tickbox questions is counted as 'no' rather than missing.*	In regression analyses:<br><br>0 = No<br>1 = Yes
**Perceived helpfulness** of AI/LLMs for mental health support (RQ2)	[Contingent on having selected 'AI/LLM platforms (e.g. ChatGPT, Gemini, Copilot' in previous question]<br><br>Based on your selection, please let us know:<br><br>How helpful was the support?	• Unhelpful<br>• Neither helpful nor unhelpful<br>• Helpful	Radio button	None	In regression analyses:<br><br>0 = 'Unhelpful' **and** 'Neither helpful nor unhelpful'<br>1 = 'Helpful'
**Perceived unmet needs** amongst those using AI/LLMs mental	In the last year, would you have liked to access additional	• Friend<br>• Family<br>• School<br>• Community or youth organisation/charity<br>• Health services and social care	Tickbox	Recoded to:<br>• Perceived unmet need (selected any additional support option)	In regression analyses:

health support (RQ3)	mental health support from any of the following?<br><br>*(Leave blank if none apply.)*	• Online/helpline<br>• Other<br>• No, I did not want additional support		• No perceived unmet need (selected 'No, I did not want additional support' or no response*)<br><br>**As we restricted our sample to those who reached this question, non-response within tickbox questions is counted as 'no' rather than missing.*	0 = No perceived unmet need<br>1 = Perceived unmet need
**Use of AI/LLM as a substitute for or as a supplement to** other forms of mental health support (RQ4)	In the last year, have you received mental health support from any of the following [category]?<br><br>*Please tick all that apply. Leave blank if not received*	**[Category: Friends and family]**<br>• Parent, step-parent or carer<br>• Sibling(s)<br>• Someone else in your family<br>• Friend(s)<br>• A family friend or another parent<br>**[School and community sources]**<br>• Form tutor<br>• Teaching Assistant<br>• Other teacher at school<br>• School nurse<br>• School counsellor/psychologist or pastoral support staff<br>• EMHP (Educational Mental Health Practitioner) or Mental health support teams<br>• Special Educational Needs Coordinator (SENCO)<br>• Other member of school staff<br>• Peer mentor at school<br>• Youth worker or mental health worker who visits the school (e.g. from a charity)<br>• Community group accessed outside of school (e.g. charity, youth club/organisation)	Tickbox	Recoded hierarchically according to 'most formal' type of support accessed:<br><br>• [If any of the health services and social care sources were ticked] → Formal support<br>• [Otherwise, if any of the school and community sources were ticked] → Semi-formal support<br>• [Otherwise, if any of the friends and family sources were ticked] → Informal support<br>• [Otherwise, if any of the digital and online services	N/A

		• Faith-based/religious contact<br>• Community mental health hubs [For Liverpool, the following example was included]: '(e.g. YPAS)<br>• [For Liverpool, the following was also]: Wellbeing clinic		were ticked] → Other online<br>• [Otherwise] → AI only	
		**[Category: Health services and social care]**<br>• GP (family doctor) or someone who works in Primary Care<br>• Child & Adolescent Mental Health Services (NHS CAMHS, Mental health support for children and young people) [For Liverpool, the following examples were included:] '(NHS CAMHS; FRESH CAMHS, Mental health support for children and young people)'<br>• Emergency Department (A&E)<br>• Link worker (either from Health or Social Care) or social prescriber<br>• NHS 111<br>• Other NHS<br>• Private counsellor/therapist<br>• Social worker/family support worker<br>• Other			
		**[Category: Digital and online sources]**<br>• Helplines (telephone, text, app, or web based - e.g. Childline, Kooth, TellMi, BetterHelp)<br>• Official informational websites or apps (e.g. NHS websites, ChatHealth)<br>• Other web-based information platforms (e.g. YouTube, blogs)<br>• Social media posts (e.g. TikTok, Instagram)<br>• Online communities (e.g. chatrooms, forums)<br>• Other<br>*[i.e. same as for RQ1, but without AI/LLMs]*			

Table S5 | Predictor variables used in the analyses

Predictor variable	Question wording	Response options	Answer type	Additional processing (if any)
		**Identity**		
**Gender**	Which of the following options best describes your gender identity? (Gender identity is how you think and feel about yourself, which may or may not differ from the way you were described at birth)	• Girl/Woman<br>• Boy/Man<br>• Gender diverse (click to expand)<br>• Don't know/Not sure<br>• Prefer not to say	Radio button	Recoded to:<br>• Girl/Woman<br>• Boy/Man<br>• Trans/Gender diverse*<br>• Don't know/Not sure<br>• Prefer not to say<br><br>**Trans/Gender diverse category derived as a category containing participants who selected 'Gender-diverse' on the first gender identity question and/or 'Yes' on the trans identity question.*
	[If 'Gender diverse (click to expand)' was chosen, the following was then presented]<br><br>If you would like, please specify further:	• Non-binary<br>• Bigender/pangender/ omnigender<br>• Genderfluid/genderflux<br>• Agender/gender neutral<br>• Multiple of these describe me<br>• None of these describe me		
	Do you identify as trans (transgender)?	• Yes<br>• No<br>• Don't know/Not sure<br>• Prefer not to say		
**Year group**	[Secondary schools] What is your year group?	• 7-13	Dropdown	Recoded to:<br>• Key Stage 3 (Year 7-9)<br>• Key Stage 4 (Year 10-11, Foundation Level, Level 1, Level 2)<br>• Key Stage 5 (Year 12-13, Level 3, A Levels, T Level)
	[Further education colleges] What are you studying?	• Foundation Level<br>• Level 1<br>• Level 2<br>• Level 3<br>• A Levels<br>• T Level		

**Ethnicity**	What is your ethnic group?	• White<br>• Mixed/Multiple Ethnic Groups<br>• Asian/Asian British<br>• Black/African/Caribbean/Black British<br>• Arab<br>• Other Ethnic Group	Tickbox	'Arab' collapsed into the 'Other Ethnic Group' in keeping with ONS Census Classification 6a.
**Self-identified neurodivergence**	Do you think you have ADHD or are autistic and/or are neurodivergent in another way?	• Yes<br>• No<br>• Not sure<br>• Prefer not to say	Radio button	None
**Mental health and self-harm**				
**Depression and anxiety**	RCADS-25	25-item validated scale comprised of an anxiety subscale (15 items) and a depression subscale (10 items) with response options Never (0); Sometimes (1); Often (2); Always (3)	Radio button	We generated an RCADS total (depression and anxiety) t-score according to scoring guidance.[1] As the guidance does not include norms for trans/gender diverse individuals, for them we apply the more conservative (i.e. higher) threshold (that for girls). This variable was subsequently scaled in order to aid with interpretability of regression coefficients.
**Deliberate self-harm**	Have you ever deliberately self-harmed (for example by taking an overdose or injuring yourself on purpose in some way)?	• Yes<br>• No<br>• Prefer not to say<br>• Not sure what this means	Radio button	None
**Social adversity**				
**Parental mental health and substance use problems**	Has your parent/carer ever had a mental health problem (like depression, anxiety, or an eating disorder)?	• Yes<br>• No<br>• Don't know<br>• Prefer not to say	Radio button	Recoded as a hierarchical composite exposure variable:<br>• [If either item = 'Yes'] → Yes<br>• [If both items = 'No'] → No<br>• [Otherwise, if either item = 'Prefer not to say'] → Prefer not to say<br>• [Otherwise, if either item = Don't know'] → Don't know
	Has your parent/carer ever had a substance use problem (this means regularly consuming large amounts of alcohol	• Yes<br>• No<br>• Don't know<br>• Prefer not to say	Radio button	

	or drugs)?			• [Otherwise] → NA
**Bullying**	Have you been bullied at school in the past couple of months?	• I have not been bullied in the past couple of months<br>• Once, twice or few times<br>• 2 or 3 times a month<br>• About once a week<br>• Several times a week	Radio button	None
**Loneliness**	How often do you feel lonely?	• Hardly ever or never<br>• Some of the time<br>• Often	Radio button	None
**Food insecurity**	At home, do you go to bed hungry because there is not enough food in the house?	• Never or hardly ever<br>• Some of the time<br>• Often	Radio button	None

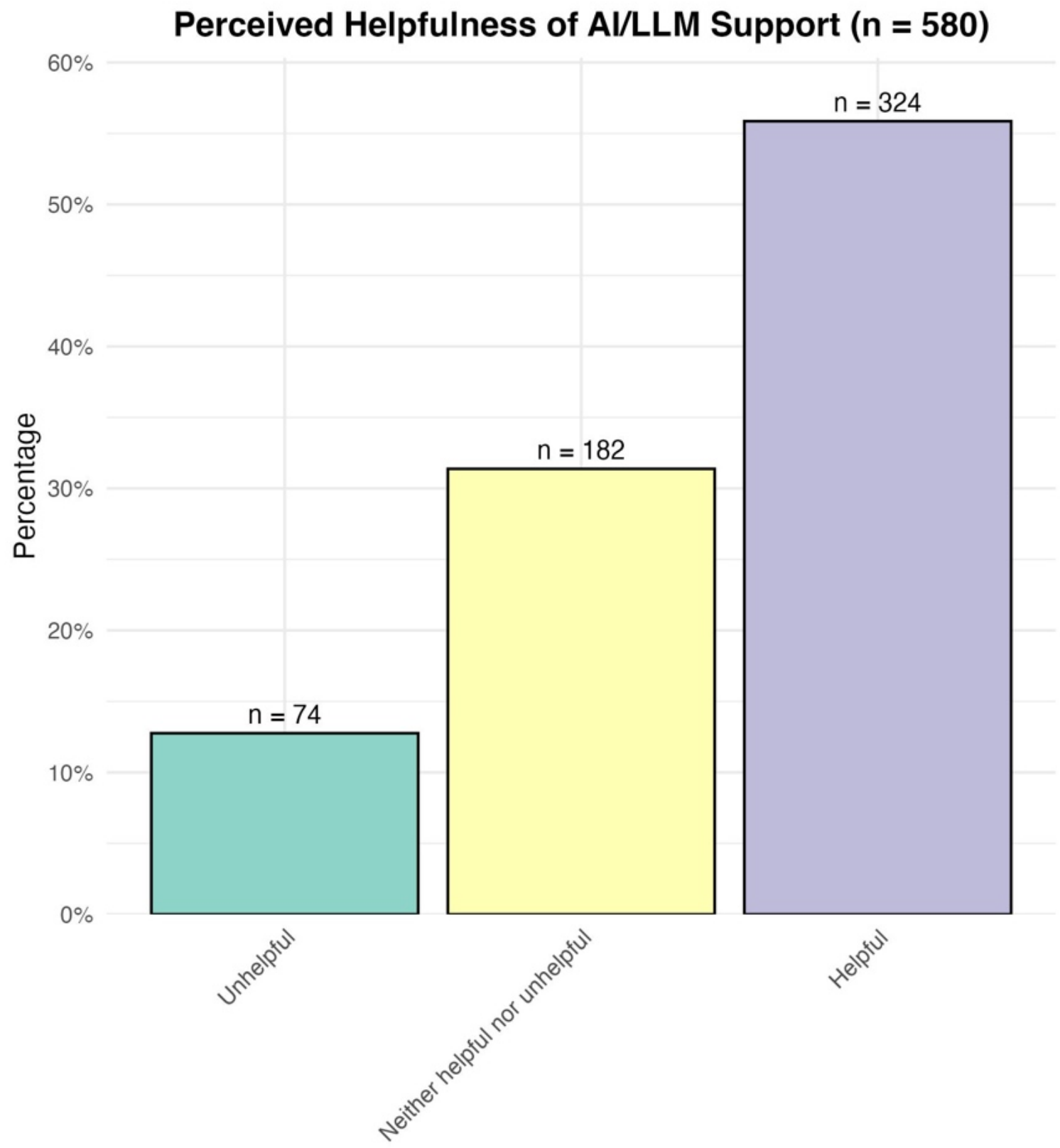

Figure S1 | Perceived helpfulness of AI/LLM support (RQ 2). *Percentages are calculated based on the total number of students with valid outcome data.*

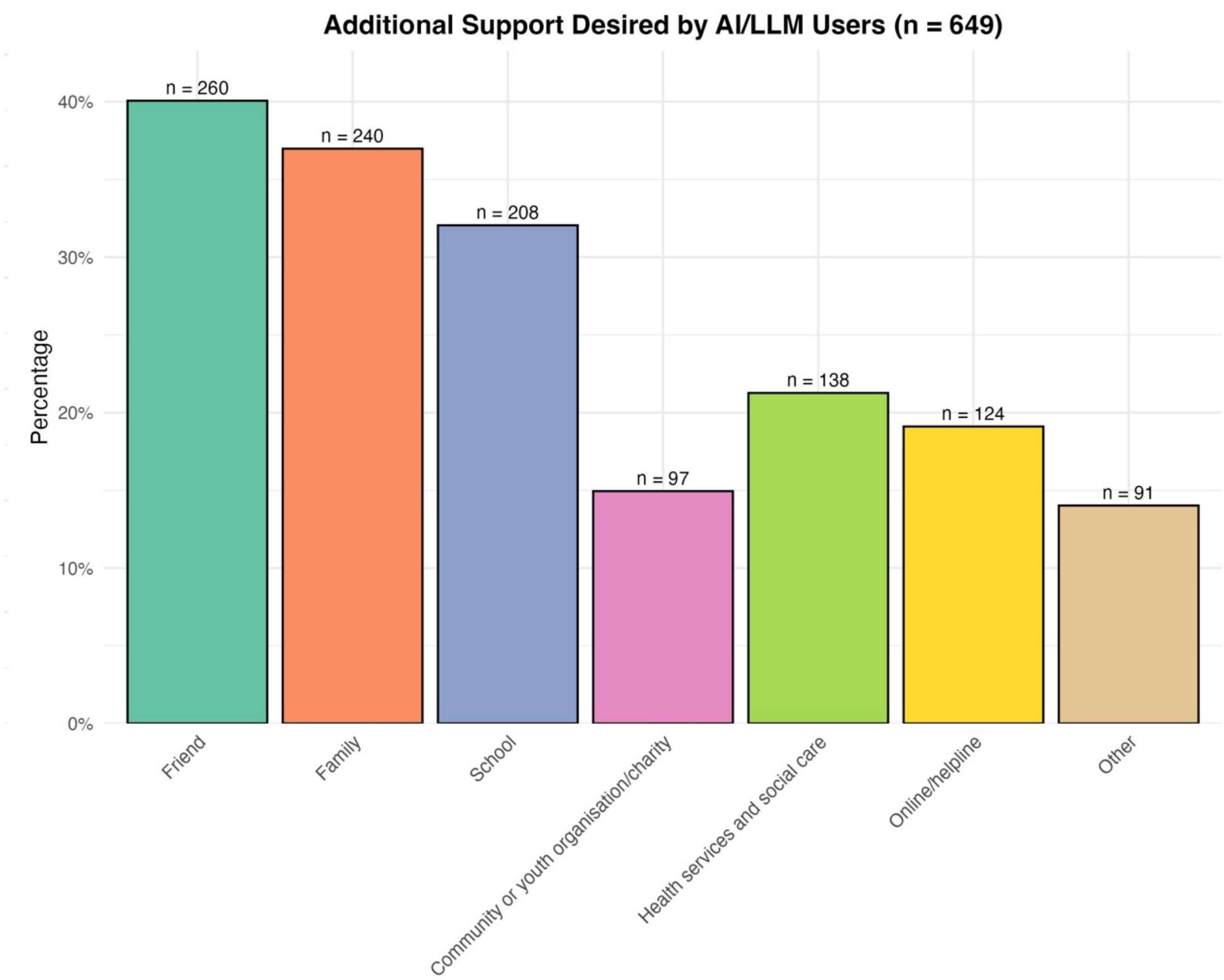

Figure S2 | Additional mental health support desired by AI/LLM users (RQ 3). *Percentages are calculated based on the total number of students with valid outcome data. Percentages add to >100% as students could tick multiple options.*

References

1. Chorpita BF. *Revised Children's Anxiety and Depression Scale: User's Guide*. 2020.
